# VALUE AND INFORMATION NEEDS FOR DEMENTIA FAMILY CAREGIVERS: CONSIDERATIONS DURING END-OF-LIFE DECISION MAKING

**DOI:** 10.1093/geroni/igz038.3494

**Published:** 2019-11-08

**Authors:** Karen E Schlag, Lorie Kmetz, David Burrows, Jung Kwak

**Affiliations:** The University of Texas at Austin, Austin, Texas, United States

## Abstract

Family caregivers often make key end-of-life care decisions for their relatives. For those caring for persons with dementia (PWDs), a third of older decedents, making end-of-life decisions as a surrogate is especially challenging. Notably, few evidence-based interventions exist to support caregivers in this capacity. Guided by the Ottawa Decision Support Framework which recognizes three determinants of informed decisions - information, value clarity, and support, the current study identifies key value considerations and information needs among family caregivers as they weigh decisions regarding hospice enrollment and artificial nutrition and hydration (AHN) for PWDs. One focus group (n=7) and four individual interviews (n=4) were conducted with dementia family caregivers. All face-to-face and telephone interviews were audio-recorded, transcribed verbatim, and verified for accuracy. Thematic analysis (Braun & Clarke, 2006) was conducted to identify and organize themes. Two main themes and subthemes emerged: 1. Caregivers expressed hospice-related values including having enough knowledge about hospice treatments for both Alzheimer’s and new symptoms, having caregiver support services, considering family needs, and weighing the extent the PWD can engage with others meaningfully and remain at home. 2. Caregivers shared AHN-related values including clearly understanding AHN treatments, services and risks for the PWD considering the patient’s functional status. Participants’ information needs reflected their priority of practical needs being met. These findings offer implications for how to design decision support tools and interventions that provide practical and specific information on the benefits and risks of hospice and AHN for PWDs and caregivers.

